# Supplementary material for: Host induced gene silencing of the Sclerotinia sclerotiorum ABHYDROLASE-3 gene reduces disease severity in Brassica napus
Source: PLoS One. 2022 Aug 26;17(8):e0261102. doi: 10.1371/journal.pone.0261102 (PMC9417021; doi:10.1371/journal.pone.0261102)
Supplement: S1 Table — (PDF) [file pone.0261102.s001.pdf]

Organism	Gene ID	Name	Percent amino acid identity to SS1G_01703
Botrytis fragariae	Bfra_002567	Alpha beta hydrolase fold protein	70%
Botrytis cinereal	BofuT4_P121410	Lipase/esterase	70%
Mollisia scopiformis	LY89DRAFT_594986	Alpha/beta-hydrolase	61%
Acephala macrosclerotiorum	BDZ45DRAFT_40961	Alpha/beta-hydrolase	58%
Phialocephala subalpine	PAC_04011	Putative sterigmatocystin biosynthesis lipase/esterase STCI	57%
Rhynchosporium commune	RC07_04596	Putative sterigmatocystin biosynthesis lipase/esterase STCI	57%
Rhynchosporium agropyri	RAG0_00765	Putative sterigmatocystin biosynthesis lipase/esterase STCI	57%
