# Supplementary material for: Host induced gene silencing of the Sclerotinia sclerotiorum ABHYDROLASE-3 gene reduces disease severity in Brassica napus
Source: PLoS One. 2022 Aug 26;17(8):e0261102. doi: 10.1371/journal.pone.0261102 (PMC9417021; doi:10.1371/journal.pone.0261102)
Supplement: S2 Table — (PDF) [file pone.0261102.s002.pdf]

Gene ID	Name
BnaA03g38630D	PR1
BnaC03g45470D	PR1
BnaA07g17230D	PR2
BnaA09g36610D	PR2
BnaA03g28770D	PR4
BnaC03g33890D	PR4
BnaA06g13430D	PR5
BnaA07g21910D	PR5
BnaA03G20310D	Basic endochitinase CHB4
BnaA05G03420D	CHI
BnaC03G24300D	Chitinase family protein
BnaC04G49090D	Endochitinase
BnaC04G53030D<br>BnaC05G01140D	CHI<br>Chitinase family protein
BnaC09G51720D	Chitinase family protein
BnaA09g07000D	Beta-1,3-glucanase
BnaA08g08720D	Beta-1,3-glucanase-like protein
BnaA01g09070D	Beta-1,3-glucanase-like protein
BnaA03g43760D	Beta-1,3-glucanase-like protein
BnaC04g24330D	Beta-1,3-glucanase 2
BnaAnng23590D	Beta-1,3-glucanase 2
BnaA09g36610D	Beta-1,3-glucanase 2
BnaA04g02500D	Beta-1,3-glucanase 2
BnaA04g02490D	Beta-1,3-glucanase 2
BnaC08g28170D	Beta-1,3-glucanase 2
BnaC08g28150D	Beta-1,3-glucanase 2
BnaA01G37310D	Feruloyl CoA ortho-hydroxylase 1
BnaC01G37530D	Feruloyl CoA ortho-hydroxylase 1
BnaA07G16850D	Probable WRKY transcription factor 70
BnaA09G35840D	Probable WRKY transcription factor 70
BnaC06G15910D	Probable WRKY transcription factor 70
BnaA01G03290D	L,L-diaminopimelate aminotransferase
BnaA03G38440D	EDTS5
BnaC03G45280D	EDTS5
BnaA02G07380D	Cytochrome P450 86
BnaA04G21360D	ABCG2
BnaA05G27640D	Glycerol-3-phosphate acyltransferase 5
BnaA07G15220D	Omega-hydroxypalmitate O-feruloyl transferase
BnaC03G12340D	Cytochrome P450 86
BnaC03G53920D	Fatty acyl-CoA reductase
BnaC04G45080D	ABCG2
BnaC05G02350D	3-ketoacyl-CoA synthase
BnaC07G49710D	Cytochrome P450 86B
BnaCNNG59260D	Omega-hydroxypalmitate O-feruloyl transferase
BnaC05g04460D	CALS7
BnaC05g04470D	CALS7
BnaA09g49880D	CALS7
BnaA10g04230D	CALS7
BnaC09g44080D	CALS3
BnaA10g20270D	CALS3
SS1G_00862 (EDN91459)	cysteine protease (calpain family)
SS1G_05329 (EDO02852)	aspartyl protease
SS1G_03282 (EDO00808)	serine protease (subtilisin-like)
SS1G_05348 (EDO02871)	metalloprotease
SS1G_05349 (EDO02872)	metalloprotease
SS1G_00891 (EDN91488)	beta-1,4-endo-glucanase
SS1G_03387 (EDO00913)	endo-glucanase
SS1G_02334 (EDN99480)	exo-glucanase (cellobiohydrolyase)
SS1G_00892 (EDN91489)	exo-glucanase
SS1G_02501 (EDN99643)	Concanavalin A-like lectin/glucanase
SS1G_01662 (EDN96736)	beta-glucosidase
SS1G_09366 (EDN93499)	beta-glucosidase
SS1G_06304 (EDO03823)	beta-glucosidase
SS1G_07146 (EDO04663)	beta-glucosidase
SS1G_05368 (EDO02891)	beta-glucosidase
SS1G_12622 (EDN97768)	beta-glucosidase
SS1G_01021 (EDN91618)	beta-glucosidase
SS1G_12191 (EDN95985)	endo-1,4-beta-xylanase
SS1G_10092 (EDN94219)	endo-beta-xylanase
SS1G_03618 (EDO01144)	endo-beta-xylanase
SS1G_00746 (EDN91343)	beta-mannosidase
SS1G_05977 (EDO03496)	beta-mannosidase
SS1G_08208 (EDN92345)	endo-1,4-beta-mannosidase
SS1G_09367 (EDN93500)	alpha-xylosidase
SS1G_04662 (EDO02186)	alpha-galactosidase
SS1G_03386 (EDO00912)	alpha-galactosidase
SS1G_02462 (EDN99605)	alpha-l-arabinofuranosidase
SS1G_03602 (EDO01128)	alpha-l-arabinofuranosidase
SS1G_13747 (EDN98888)	DCL1
SS1G_13161(EDN98303)	RDRP2
SS1G_03377 (EDO00903)	RDRP 3
SS1G_00334 (EDN90934)	AGO2
SS1G_05990 (EDO03509)	lipase/esterase
SS1G_00877 (EDN91474)	extracellular lipase
SS1G_01803 (EDN96777)	alpha beta-hydrolase (esterase/lipase)
SS1G_02163 (EDN99310)	alpha beta-hydrolase (esterase/lipase)
