# Supplementary material for: Host induced gene silencing of the Sclerotinia sclerotiorum ABHYDROLASE-3 gene reduces disease severity in Brassica napus
Source: PLoS One. 2022 Aug 26;17(8):e0261102. doi: 10.1371/journal.pone.0261102 (PMC9417021; doi:10.1371/journal.pone.0261102)
Supplement: S3 Table — (PDF) [file pone.0261102.s003.pdf]

Name	Fwd. Primer	Rev. Primer
SS1G_01703 cloning (KpnI/XhoI)	GTATAGGTACCTTCTGCCGGAAACCCTCTTC	GTATACTCGAGACCGCCGATTGTGAAGACTT
SS1G_01703 genotyping	TTCTGCCGGAAACCCTCTTC	ACCGCCGATTGTGAAGACTT
sRNA1 hairpin	ACGACATGATGAGTTCTGAAGGCCTTTCGATTCCGAACGGATCC	
