# Supplementary material for: Host induced gene silencing of the Sclerotinia sclerotiorum ABHYDROLASE-3 gene reduces disease severity in Brassica napus
Source: PLoS One. 2022 Aug 26;17(8):e0261102. doi: 10.1371/journal.pone.0261102 (PMC9417021; doi:10.1371/journal.pone.0261102)
Supplement: S4 Table — (PDF) [file pone.0261102.s004.pdf]

Name	Fwd. Primer	Rev. Primer	Efficiency (%)
SS1G_12350	CGATACTGTGCCTGTGACCA	CCTCTCCTCAAGCGCCATAG	102
SS1G_01703	CTTCCATCCCTGCCGCTTAC	CCATAGCGGCTCGATCTAGAATC	99
SS18S rDNA	AGCCGATGGAAGTTTGAGGC	CTCGTTGGCTCTGTCAGTGT	103
BNATGP4	CGTCTTCCTCTTCCCTCACC	ACAGTTGGAATAGAATAGTAGGCTC	100
BNHMG I/Y	GGTCGTCCTCCTAAGGCGAAAG	CTTCTTCGGCGGTCGTCCAC	95
BNPR1	TGTGGCAAAGCAAGGTGTAA	TTCCCCGAGGATCATAGTTG	98
BNDCL2A	TGAGAAACGGCATGAGGTTCA	GTGAAGGTTGTTATGCAGCGT	92
sRNA1	ACGACATGATGAGTTCTG	AGAGGTAGTAGGTTGT	96
