# Supplementary figures and images for: Host induced gene silencing of the Sclerotinia sclerotiorum ABHYDROLASE-3 gene reduces disease severity in Brassica napus
Source: PLoS One. 2022 Aug 26;17(8):e0261102. doi: 10.1371/journal.pone.0261102 (PMC9417021; doi:10.1371/journal.pone.0261102)

BN1703 genotyping

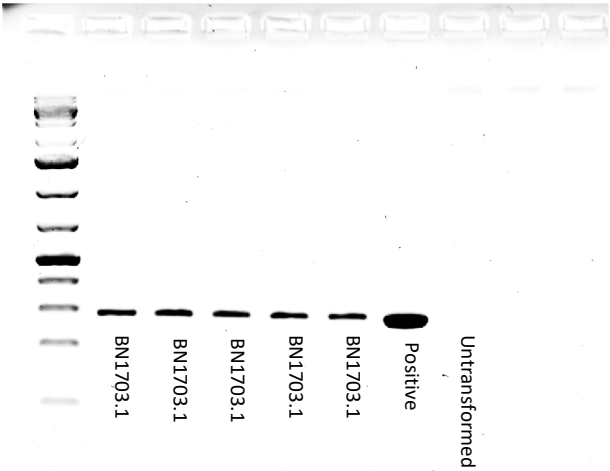

Supplement: S1 Fig — Positive control uses the 1703:pHELLSGATE8 construct. (PDF) [file pone.0261102.s005.pdf]

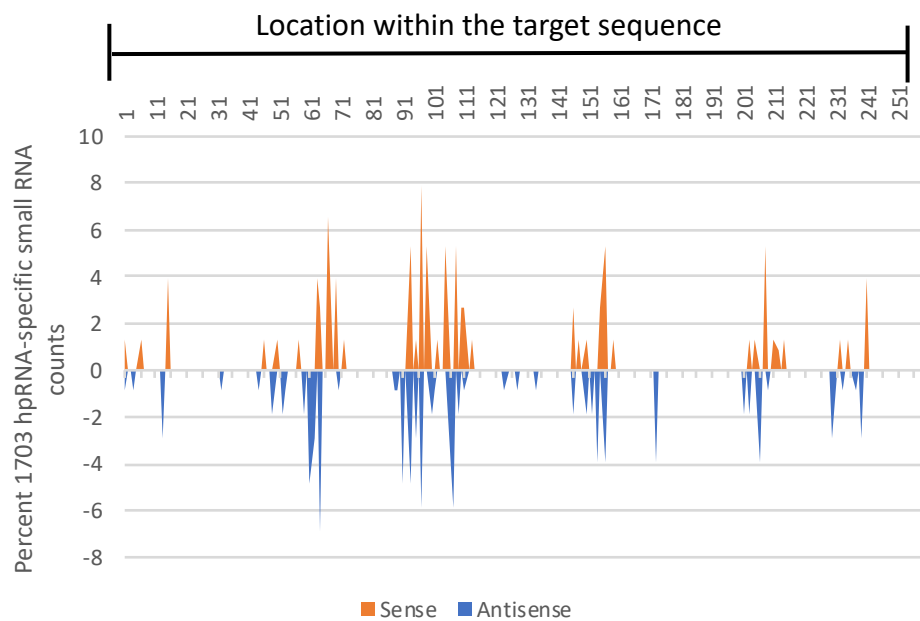

Supplement: S2 Fig — Reads were plotted to their respective location upon the transgene sequence using Geneious Prime software (https://www.geneious.com) to identify highly abundant molecules. (PDF) [file pone.0261102.s006.pdf]

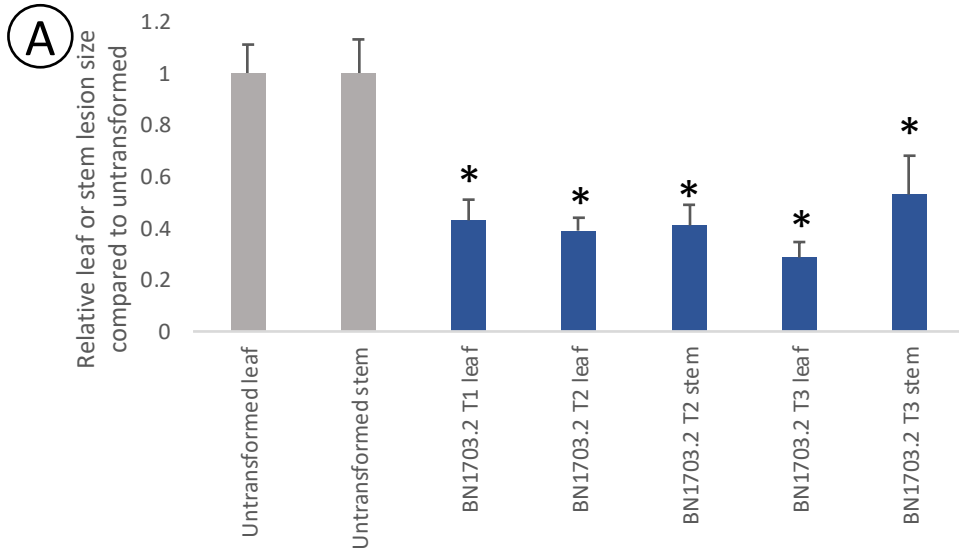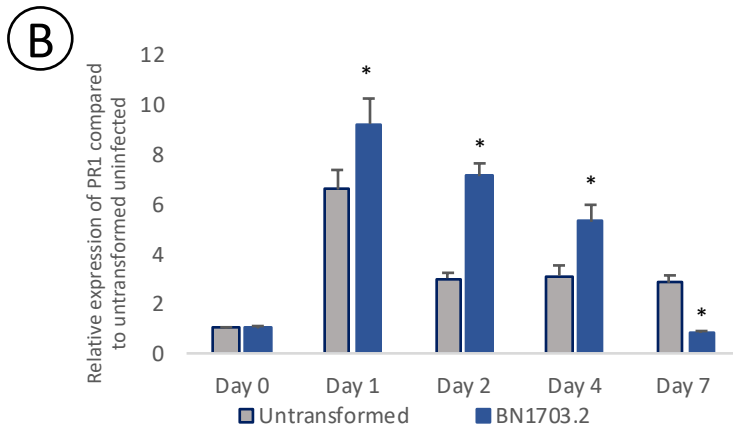

Supplement: S3 Fig — (a) Asterisks represent statistical differences from the untransformed control (one-tailed t-test with Bonferroni correction, p<0.05). (b) Samples were normalized to the housekeeping reference BNATGP4. Data represents 3 biological replicates with error bars representing standard error. Asterisks represent statistical differences from the untransformed control (one-tailed t-test with Bonferroni correction, p<0.05). (PDF) [file pone.0261102.s007.pdf]

A

*B. napus*

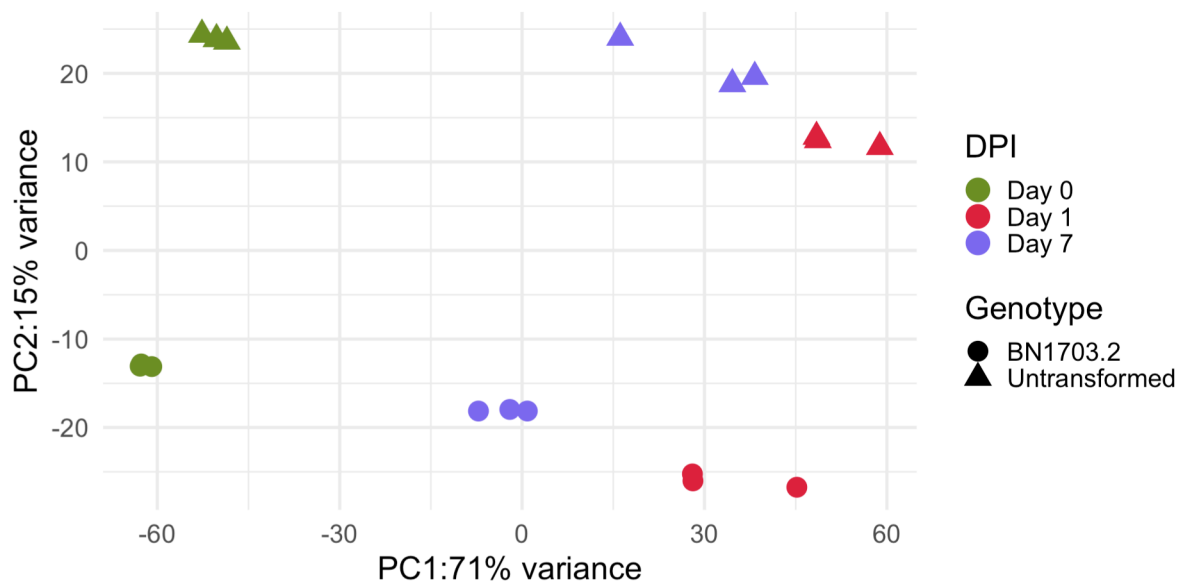

B

*S. sclerotiorum*

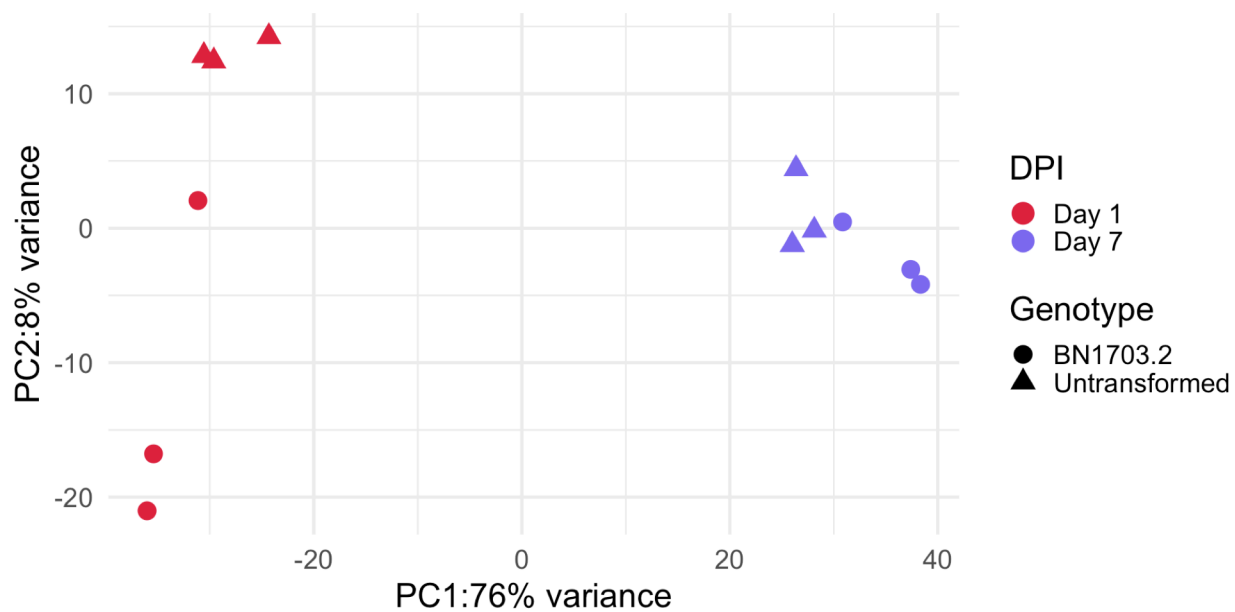

Supplement: S4 Fig — (PDF) [file pone.0261102.s008.pdf]

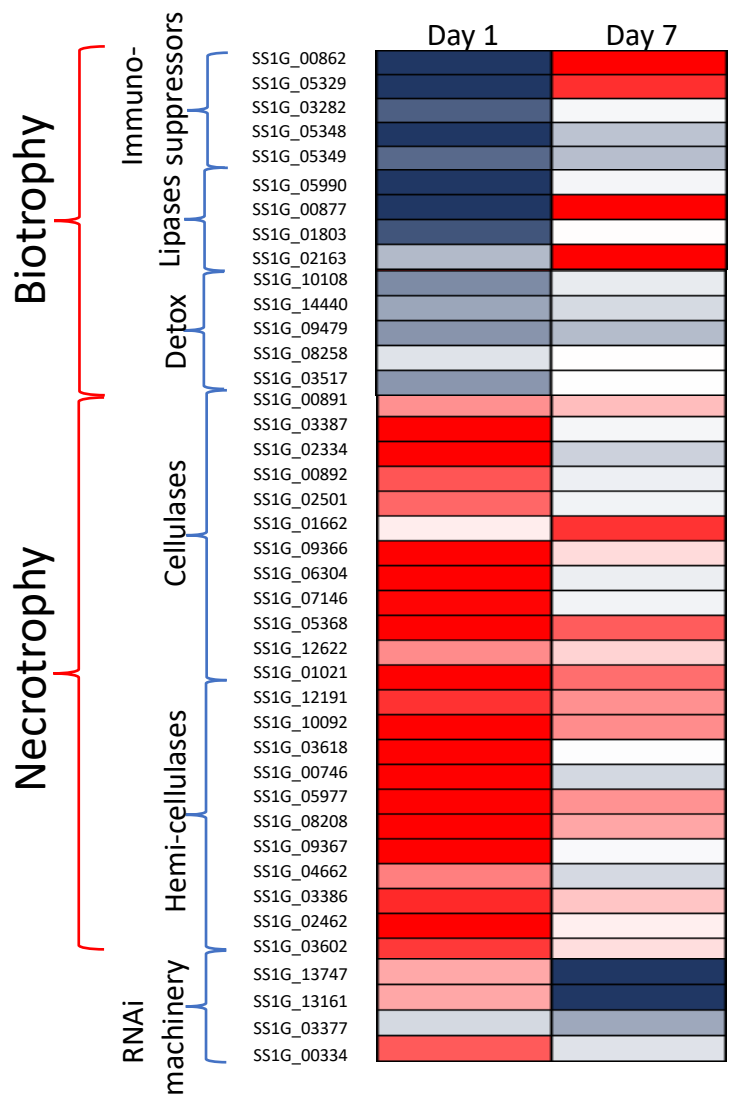

Log<sub>2</sub> fold change (BN1703.2 / untransformed)

-2      0      2

Supplement: S5 Fig — Genes were involved in S. sclerotiorum infection common to the biotrophic and necrotrophic stages of infection (false discovery rate<0.05). (PDF) [file pone.0261102.s009.pdf]

ZY821 Day 7

BN1703.2 Day 7

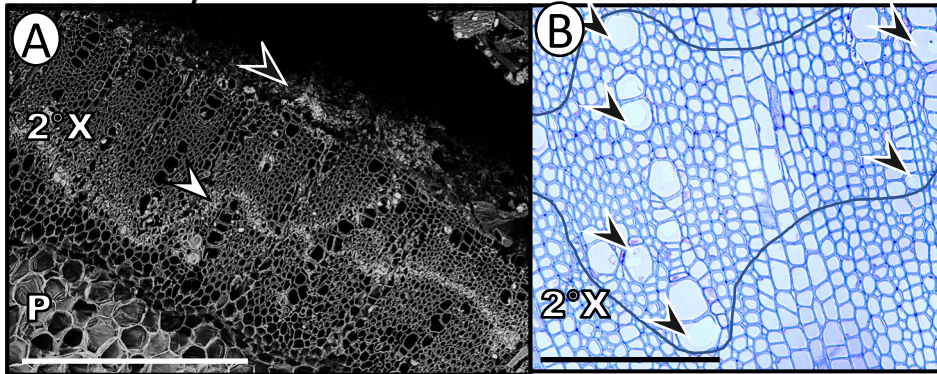

Untransformed Uninfected BN1703.2 Uninfected

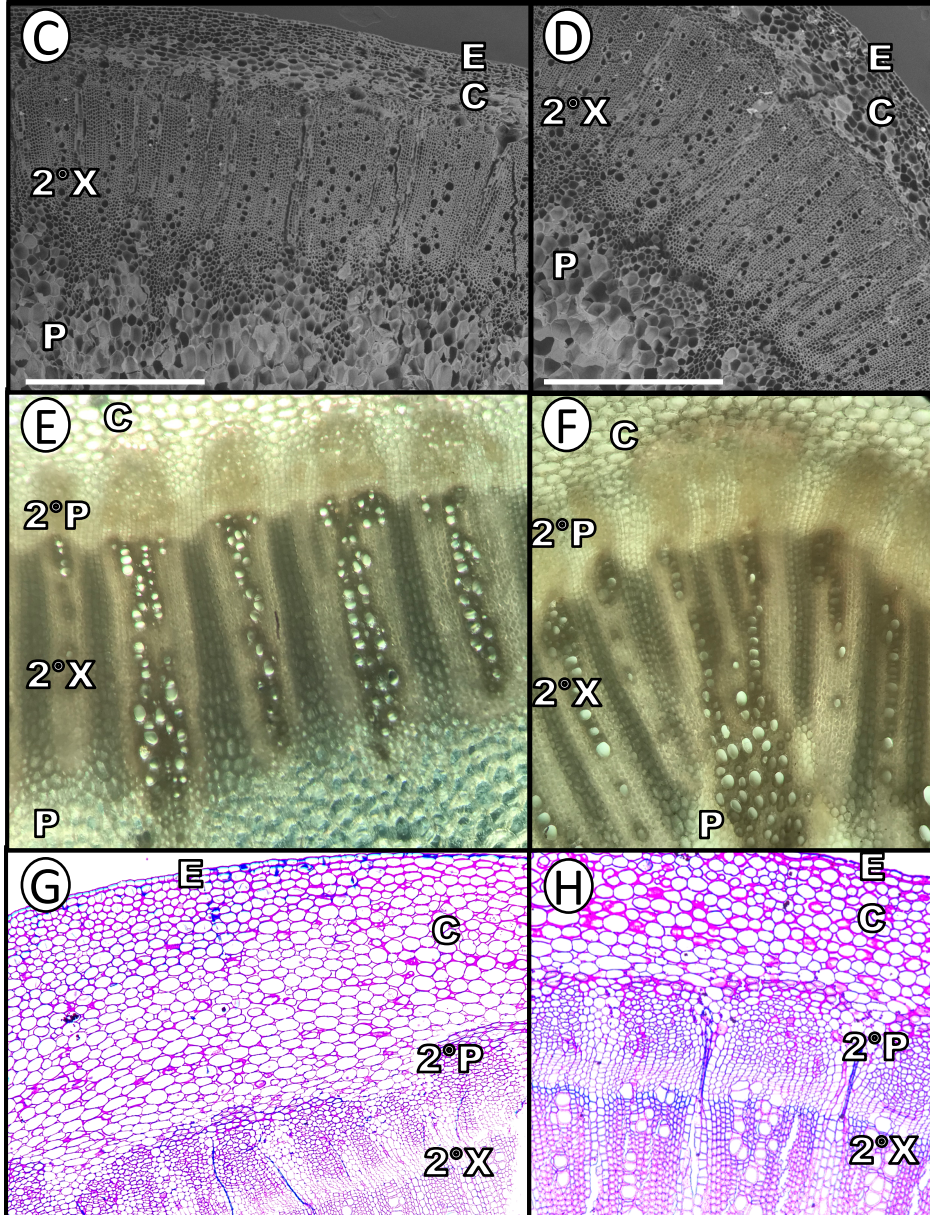

Supplement: S6 Fig — (A) Scanning electron micrograph of a stem cross-section of the semi-resistant cultivar of Brassica napus cv. ZhongYou 821 7 days post-inoculation. Black arrowheads indicate S. sclerotiorum hyphae and white indicates the vascular coating. Scale bar represents 0.5 mm. (B) Toludine-blue and Periodic-Acid Schiff’s staining of BN1703.2 infected stem cross-sections 7 days post inoculation. Black arrowheads indicate negative detection to the vascular coating deposits within the secondary xylem by these stains. Scale bar represents 50 μm. Scanning electron micrographs of uninfected untransformed (C) and BN1703.2 (D) stem cross-sections. Scale bars represent 0.5 mm. Hand-cut stem transverse sections stained with Sudan IV of uninfected untransformed (E) and BN1703.2 (F) stems. Uninfected stems exhibit low affinity for Sudan IV in both. Scale bars represent 250 μm. Uninfected untransformed (G) and BN1703.2 (H) stem cross-sections stained with aniline blue and periodic acid-Schiff’s reagent. Aniline blue staining is scant in both cultivars, indicating low callose deposition. Scale bars represent 250 μm. (PDF) [file pone.0261102.s010.pdf]
